# Special Issue “Advances in Epitaxial Materials”-Editorial Preface

**DOI:** 10.3390/ma13112622

**Published:** 2020-06-09

**Authors:** Mike Leszczynski

**Affiliations:** Institute of High Pressure Physics, Polish Academy of Sciences, Sokolowska 29/37, 01-142 Warsaw, Poland; mike@unipress.waw.pl

**Keywords:** epitaxy, semiconductors, optoelectronics, electronics

## Abstract

In this Special Issue, we have 10 excellent papers on epitaxy. In this editorial preface, I will make comments on the following issues: (1) applications of the materials examined, (2) lattice mismatch, (3) epitaxial growth methods used, (4) characterization methods used, (5) material problems: solved and still to be solved. The “Advances in Epitaxial Materials” has a big advantage of having, in one issue, papers on different materials, but in every paper the reader should find interesting information on epitaxial growth and characterization.

## 1. Applications of the Materials Examined

All ten papers deal with materials for electronics, which reflects the situation that one can rarely find other applications of epitaxial structures. Many epitaxial technologies, such as Si-Ge, are mature enough that they are very rarely mentioned in the scientific papers. The properties of others are still being intensively explored. Half of the papers in this issue deal with nitride semiconductors. This is not surprising, as the present situation in this field of science and technology is often described half-jokingly as “GaNification” because gallium nitride and its related compounds revolutionize many areas of life. White LEDs based on blue LEDs illuminating phosphor have created a market worth tens of billions of euros. Recent advances in deep-UV LEDs have created a bright future for the use of these devices in sterilization and disinfection. RGB (red based on arsenides/phosphides semiconductors, green, blue based on nitrides) projectors based on LEDs or Laser Diodes (LDs) push out other projectors from the market. Lighting, displays, Li-Fi telecommunication, and many other optoelectronic applications of nitrides take advantage of the high luminosity of nitrides. Their wide band-gap and high stability make them a perfect material for high-power and high-frequency transistors already present in the market.

However, one should be aware of the fact that nitrides are very difficult to grow and process.

The popularity of nitride semiconductors is reflected in the Special Issue in papers of R. Czernecki et al. [1], Y.L. Casallas-Moreno et al. [2], M. Sarzynski et al. [3], Akira Kusaba et al. [4], and Takeshi Ohgaki et al. [5]. 

Two papers describe the growth and properties of more classical III-V epitaxial layers: GaInAsSb on GaAs [6] and InAlAs/InGaAs/InP [7]. These semiconductors are used in manufacturing high-mobility transistors, photovoltaic cells, and infrared/red light emitters, as well as novel devices as cascade lasers.

Photovoltaic applications are represented by polymorphism-based MOF (metalorganic frameworks) [8]. Many electronic devices, as memories, thermochromic smart windows, or Mott field effect transistors, are based on vanadium oxide, as described by Yuanjun Yang [9]. 

In the electromagnetic spectrum, there is still a gap in the THz region in both the emitters and the detectors. As THz devices are very desirable in many applications, such as cancer diagnosis, food inspection, explosive detection, electronics, and communications, there are many groups working in that field. In this Special Issue, Kazuhiro Endo et al. [10] report on the growth and properties of BiSrCeCuO layers on SrTiO_3_ and LaAlO_3,_ which are promising materials for THz devices.

## 2. Lattice Mismatch 

All epitaxial layers described in this Special Issue are highly mismatched to the substrates. Therefore, this issue is discussed in almost all of the papers. The most spectacular result in reducing the threading dislocation density for the highly mismatched GaInAsSb on GaAs was obtained by Qi Lu [6] who used strained GaInSb/GaSb multiple quantum wells as dislocation filtering layers. This approach can be used for other epitaxial systems.

An increasingly popular method of epitaxy is growing the two-dimensional object as quantum wires or stripes. In this case, the strain can be accommodated by elastic deformation. This issue is discussed in the paper of Y.L. Casallas-Moreno et al. [2] for InN nanocolumns on AlN/Si substrates and of M. Sarzynski et al. [3] for InGaN quantum wells on narrow stripes made on GaN substrates.

## 3. Epitaxial Growth Methods 

In the Special Issue, four papers [1,3,4,10] report epitaxial growth using Metalorganic Chemical Vapor Phase Epitaxy (MOVPE). This is the most popular method used for growing most epitaxial layers in the semiconductor industry. However, in this method, several growth parameters (temperature, pressure, flows of reactant and carrier gases) influence the properties of the layers. These parameters are not independent of each other which makes any growth optimization extremely difficult. In the paper of Czernecki et al. [1], the additional growth parameter is discussed: the distance of the showerhead to the sample. The experimental results show the importance of reactor design, and this issue is very rarely taken into account.

The second epitaxial method used mostly in academic labs is molecular beam epitaxy (MBE). This method was also used in four papers [2,5,6,7]. Such a balance between the number of papers on MOVPE and MBE reflects the interests of scientists. In industry, MOVPE is likely to dominate by more than one order of magnitude. Two other papers used Liquid Phase Epitaxy [8] and magnetron sputtering [10]. Both techniques are much cheaper than MOVPE and MBE, and may find, in future, some niche applications.

## 4. Characterization Methods Used

In almost all of the papers of the Special Issue, the High Resolution X-ray Diffraction (XRD) was used for the characterization of samples. The 2theta/omega and reciprocal lattice maps are reported. An issue not discussed in the papers is the difficulty in the interpretation of the XRD data for poor-crystallographic-quality layers. In particular, the comparison of the simulations conducted for the perfect layers and the experimental data obtained for imperfect layers must be performed with great care.

The second characterization method applied is photoluminescence (PL). In this case, the interpretation of the experimental data is not easy. For example, if one examines InGaN quantum wells, the PL peak position, its half-width, and intensity depend not only on indium content and well width, but also on indium segregation, internal electric fields, and the presence of point and extended defects. 

## 5. Material Problems: Solved and Still to be Solved

All ten papers of this Special Issue significantly contribute to the knowledge on the epitaxial growth of electronic materials. Some of them propose materials which should be considered as candidates for new electronic devices. 

However, one should be aware of the fact that—in none of the papers—point defects were taken into account. These defects strongly influence not only optical and electrical properties but also mechanical properties (lattice mismatch relaxation). The examination of point defects is extremely difficult as very few analytical methods can give information on these defects. However, breakthroughs in electronics will happen when the nature of point defects is learnt.
